# Multi-compartment analysis of the complex gradient-echo signal quantifies myelin breakdown in premanifest Huntington's disease

**DOI:** 10.1016/j.nicl.2021.102658

**Published:** 2021-04-05

**Authors:** Chiara Casella, Elena Kleban, Anne E. Rosser, Elizabeth Coulthard, Hugh Rickards, Fabrizio Fasano, Claudia Metzler-Baddeley, Derek K. Jones

**Affiliations:** a Cardiff University Brain Research Imaging Centre (CUBRIC), School of Psychology, Cardiff University, Maindy Road, Cardiff, CF 24 4HQ, UK; b Department of Neurology and Psychological Medicine, Hayden Ellis Building, Maindy Road, Cardiff CF24 4HQ, UK; c School of Biosciences, Cardiff University, Museum Avenue, Cardiff CF10 3AX, UK; d Clinical Neurosciences, University of Bristol, Bristol BS10 5NB, UK; e Birmingham and Solihull Mental Health NHS Foundation Trust, 50 Summer Hill Road, Birmingham B1 3RB, UK; f Institute of Clinical Sciences, College of Medical and Dental Sciences, University of Birmingham, Edgbaston, Birmingham B15 2TT, UK; g Siemens Healthcare Ltd, Camberly, UK; h Siemens Healthcare GmbH, Erlangen, Germany

**Keywords:** CAG, cytosine, adenine, and guanine, CC, corpus callosum, CV, coefficient of variation, DBS, disease burden score, DCL, diagnostic confidence level, DT-MRI, diffusion tensor magnetic resonance imaging, FDM, frequency difference mapping, HD, Huntington’s disease, HTT, huntingtin, mGRE, multi-echo gradient-recalled echo, MoCA, Montreal Cognitive Assessment, MTR, magnetization transfer ratio, MW, myelin water, MWF, myelin water fraction, MWI, myelin water imaging, PCA, principal component analysis, PEBL, Psychology Experiment Building Language (PEBL), RF, radiofrequency, TE, echo time, TMS, total motor score, TOPFUK, Test of Premorbid Functioning - UK Version, TR, repetition time, UHDRS, Unified Huntington’s Disease Rating Scale, WAIS-R, Wechsler Adult Intelligence Scale-Revised, WM, white matter, Frequency difference mapping, Three-pool model, Premanifest Huntington’s disease, Myelin, Executive function

## Abstract

White matter (WM) alterations have been identified as a relevant pathological feature of Huntington’s disease (HD). Increasing evidence suggests that WM changes in this disorder are due to alterations in myelin‐associated biological processes. Multi-compartmental analysis of the complex gradient-echo MRI signal evolution in WM has been shown to quantify myelin *in vivo*, therefore pointing to the potential of this technique for the study of WM myelin changes in health and disease. This study first characterized the reproducibility of metrics derived from the complex multi-echo gradient-recalled echo (mGRE) signal across the corpus callosum in healthy participants, finding highest reproducibility in the posterior callosal segment. Subsequently, the same analysis pipeline was applied in this callosal region in a sample of premanifest HD patients (n = 19) and age, sex and education matched healthy controls (n = 21). In particular, we focused on two myelin-associated derivatives: i. the myelin water signal fraction (f_m_), a parameter dependent on myelin content; and ii. The difference in frequency between myelin and intra-axonal water pools (Δω), a parameter dependent on the ratio between the inner and the outer axonal radii. f_m_ was found to be lower in HD patients (β = −0.13, p = 0.03), while Δω did not show a group effect. Performance in tests of working memory, executive function, social cognition and movement was also assessed, and a greater age-related decline in executive function was detected in HD patients (β = −0.06, p = 0.006), replicating previous evidence of executive dysfunction in HD. Finally, the correlation between f_m_, executive function, and proximity to disease onset was explored in patients, and a positive correlation between executive function and f_m_ was detected (r = 0.542; p = 0.02). This study emphasises the potential of complex mGRE signal analysis for aiding understanding of HD pathogenesis and progression. Moreover, expanding on evidence from pathology and animal studies, it provides novel *in vivo* evidence supporting myelin breakdown as an early feature of HD.

## Highlights

• Gradient-echo data were acquired from premanifest HD patients in the callosum at 7 T.
• Reproducibility of multi-compartment analysis across callosal areas was assessed.
• Reduced myelin water signal fraction (f_m_) in HD patients suggested myelin breakdown.
• Executive function correlated with callosal f_m_ values in HD patients.
• The reported approach may aid understanding of HD pathogenesis and progression.

## 1 Introduction

### 1.1 Why study myelin changes in Huntington’s disease?

Huntington’s disease (HD) is a debilitating genetic disorder caused by an expansion of the CAG (cytosine, adenine, guanine) repeat within the huntingtin (*HTT*) gene, and characterised by motor, cognitive and psychiatric symptoms associated with neuropathological decline. Although clinical onset of the disease is formally identified with the development of movement symptoms (Nopoulos, 2016), critical pathogenic events are present early on in the disease course (see Casella et al., 2020 for a review). Accordingly, subtle and progressive white matter (WM) alterations (Dayalu and Albin, 2015), have been observed early in HD progression, before the onset of motor symptoms (Aylward et al., 2011, Bourbon-Teles et al., 2019, Ciarmiello et al., 2006, De Paepe et al., 2019, Dumas et al., 2012, Faria et al., 2016, Gregory et al., 2018, McColgan et al., 2017, Paulsen et al., 2008, Ruocco et al., 2008, Shaffer et al., 2017, Tabrizi et al., 2009, Wu et al., 2017, Zhang et al., 2018).

An increasing body of research suggests that WM changes in HD are due to changes in myelin-associated biological processes at the cellular and molecular level (Ferrari Bardile et al., 2019, Gomez-Tortosa et al., 2001, Huang et al., 2015, Jin et al., 2015, Myers et al., 1991, Radulescu et al., 2018, Teo et al., 2016, Yin et al., 2020) – for a critical review of such changes see Casella et al., (2020). Myelin, a multi-layered membrane sheath wrapping axons, is crucial for axonal structure and WM functionality (Martenson, 1992). In HD, myelin changes are suggested to follow both a topologically selective and temporally specific degeneration, with early myelinated fibres being the most susceptible to, and the first to be affected by, myelin breakdown (Bartzokis et al., 1999, 2007; Dumas et al., 2012, Faria et al., 2016, Phillips et al., 2013, Tabrizi et al., 2011).

The assessment of early myelin changes in the HD brain is therefore of fundamental importance for the understanding of disease pathogenesis and progression. Notably, as no disease-modifying treatment currently exists for HD, understanding the biological underpinning of HD-associated WM changes may prove useful for the identification of disease-related biomarkers, and for measuring responsiveness to pharmaceutical and other therapeutic approaches. Critically, given the certainty of onset in those that inherit the HD mutation, we can examine HD-associated myelin-related changes from the earliest, premanifest disease stages, with the potential to identify novel treatment targets for delaying disease onset.

### 1.2 Probing myelin changes in the HD brain with a multi-echo gradient-recalled echo (mGRE) sequence

Quantitative MRI of myelin affords valuable insight into myelin alterations and is thus of particular interest in the study of myelin-related disorders. Most neuroimaging studies that have quantified WM tissue properties in HD have used diffusion tensor magnetic resonance imaging (DT-MRI) (see Casella et al. 2020 for a review). However, while sensitive, DT-MRI measures are not specific to WM sub-compartments, challenging the interpretation of any observed change in these indices (Beaulieu, 2002, De Santis et al., 2014, Wheeler-Kingshott and Cercignani, 2009).

Other MRI techniques have the promise to provide much more myelin-specific information (MacKay and Laule, 2016). For example, myelin water imaging (MWI) quantifies the fraction of the faster decaying signal from water trapped between myelin lipid bilayers (MacKay et al., 1994), the so-called myelin water fraction (MWF). MWF has a good correlation with histological measurements of myelin, demonstrating its potential as an *in vivo* myelin marker (Laule et al., 2004, Laule et al., 2006, Webb et al., 2003). MWI techniques are typically based on spin-echo (MacKay et al., 1994) or mGRE sequences (Du et al., 2007). Interestingly, mGRE enables further characterisation of the myelin sheath by exploring its interaction with the magnetic field B_0_, which is suggestively dependent on the g-ratio (i.e. the ratio of the inner-to-outer diameter of a myelinated axon) (Wharton and Bowtell, 2012).

A plethora of studies have demonstrated the non-mono-exponential nature of mGRE signal evolution with echo time (TE) in WM (e.g. Sati et al., 2013, Wharton and Bowtell, 2012, Wharton and Bowtell, 2013), arising from sub-voxel microstructure, with distinct signal components originating from water confined to the myelin, intra-axonal and extra-axonal water pools (Cronin et al., 2017, Nam et al., 2015b, Nunes et al., 2017, Sati et al., 2013, Tendler and Bowtell, 2019, Thapaliya et al., 2018, Wharton and Bowtell, 2012). As a result of the rapid T_2_* decay of the myelin water signal, the frequency of the total signal changes with TE, producing a local, microstructure-dependent contribution to the signal phase. However, in order to uncover the specific effects of microstructure on phase signal evolution, it is necessary to remove TE-dependent signal inhomogeneities resulting from non-local field variation, together with other non-TE-dependent phase changes, such as those due to radiofrequency interaction with the tissue (Schweser et al., 2011).

For this purpose, frequency difference mapping (FDM) has been presented recently as a phase-processing technique (Kleban et al., 2021, Sati et al., 2013, Schweser et al., 2011, Tendler and Bowtell, 2019, van Gelderen et al., 2012, Wharton and Bowtell, 2013). FDM is performed by comparing frequency maps acquired at short and long TEs so as to yield local frequency difference values which depend solely upon the underlying tissue microstructure, and in particular upon the local nerve fibre orientation with respect to the applied magnetic field. Critically, since both compartmentalization and myelination are prerequisites for the generation of frequency differences, FDM has great potential for the study of myelin changes in WM (Li et al., 2016, Wisnieff et al., 2015).

The aim of the present study was to exploit, for the first time in the HD literature, the sensitivity of the complex mGRE signal to WM microstructure, and particularly to myelin content, to assess callosal myelin changes at the premanifest stage of the disease. The corpus callosum (CC) is the largest WM fibre tract in the brain and carries information between the hemispheres; additionally, this tract plays an integral role in relaying sensory, motor and cognitive information between homologous cortical regions (Aboitiz et al., 1992), and provides vital connections to cortical areas known to be affected in HD (Crawford et al., 2013). Crucially, given its perpendicular orientation with respect to the B_0_ field, as a ‘proof of concept’ of the utility of the FDM approach in HD, the CC is a natural choice. Investigating this tract indeed afforded the largest possible frequency offsets in the myelin and axonal compartments, thus giving the most marked frequency difference ‘signature’ of myelin (Sati et al., 2013, Wharton and Bowtell, 2012, Yablonskiy et al., 2014).

Previous evidence from DT-MRI and volumetric studies has shown that changes in macro- and microstructure are detectable in the CC early in the disease course (Crawford et al., 2013, Di Paola et al., 2012, Di Paola et al., 2014, Phillips et al., 2013). The aim of the present work was to provide novel evidence on callosal changes in HD, and specifically to move beyond the existing literature by employing ultra-high field susceptibility measurements in order to afford a more biologically-meaningful interpretation of microstructure changes in this tract. Importantly, scanning participants at ultra-high field strength (i.e., 7 T) afforded higher signal-to-noise ratio (SNR) and signal contrast (MacKay and Laule, 2016) per unit time, compared to more commonly-available field strengths (e.g. 3 T).

Specifically, we sought to: i. establish the reliability of this method by investigating the anatomical variability in the reproducibility of FDM across the callosum at 7 T; ii. compare two myelin-related parameters between premanifest HD patients and healthy controls; and iii. assess brain-function relationships in patients by exploring correlations between myelin content and cognitive function, as well as proximity to disease onset. The two myelin-sensitive metrics we assessed were: i. the myelin water signal fraction (f_m_) and ii. the difference in frequency offsets between myelin water pool and axonal water pool (Δω). The former is linked to the myelin volume fraction and may be used as a proxy for tissue myelin content (Laule et al., 2008, Li et al., 2015); the latter depends on the magnetic susceptibility difference and on the g-ratio, which is the ratio of the inner to outer diameter of myelinated axons (Wharton and Bowtell, 2012). Cognitive tests were selected in order to capture functioning across executive functions, working memory, social cognition and motor performance (Table 5), as these represent the earliest cognitive indicators of HD (Paulsen, 2011), and impaired performance in these domains has been associated with callosal microstructure changes (Kennedy and Raz, 2009, Lenzi et al., 2007, McDonald et al., 2018).

## 2 Materials and methods

### 2.1 Subjects

• Reproducibility study

To investigate the anatomical variability in the precision of the complex mGRE signal and the subsequent multi-compartmental analysis across the CC, six healthy subjects without known neurological or psychiatric conditions (3 female, 26–33 years-old) were scanned five times over a two-week period each. The study was approved by the Cardiff University School of Psychology Ethics Committee and written informed consent was obtained from all participants.

• HD study

For the assessment of callosal myelin content in premanifest HD, MRI scans and cognitive tests were performed on 19 premanifest HD patients and 21 age, sex, and education matched healthy controls (Table 1). The study was performed with ethics approval by the local National Health Service (NHS) Research Ethics Committee (Wales REC 5 18/WA/0172); all participants provided written informed consent.

**Table 1:** Summary of participants’ demographic and clinical background information. Age is displayed in years. MoCA = Montreal Cognitive Assessment out of 30; the higher the score the better the performance. TOPF-UK IQ = verbal IQ estimate based on the Test of Premorbid Functioning, UK version.

Group	Gender male/female (%)	Mean age (SD, range)	Mean MoCA score (SD, range)	Mean TOPF-UK IQ (SD, range)
HD (n = 19)	12(63.15)/7(36.85)	41.61 (13.1, 21–70)	27.82 (2.29, 24–30)	117.19 (11.58, 98–137.4)
Controls (n = 21)	10(47.6)/11(52.4)	45.14 (12.5, 27–71)	28.16 (2.00, 26–30)	123.42 (7.85, 109–131.9)

HD patients were recruited from the Cardiff HD Research and Management clinic, the Bristol Brain Centre at Southmead Hospital, and the Birmingham HD clinic at the Birmingham and Solihull NHS Trust. Healthy controls were recruited from Cardiff University, the School of Psychology community panel, and from patients’ spouses or family members.

In order to take part in the study, HD carriers had to be at the premanifest disease stage, and hence have no motor diagnosis, and to be enrolled in the EHDN Registry/ENROLL study (NCT01574053, https://enroll-hd.org). The progression of symptoms in ENROLL-HD participants is monitored longitudinally. As such, a full clinical dataset including medical history is available for each research participant, and some of these data were used for this study.

Table 1 summarizes information about demographic variables and performance in the Montreal Cognitive Assessment (MoCA) (Nasreddine et al., 2005) and in the Test of Premorbid Functioning - UK Version (TOPF-UK) for patients and controls. Although the two groups did not differ significantly in age, MoCA score, or TOPF-UK IQ, controls were on average slightly older and had a slightly higher IQ. Table 2 summarizes patients’ background clinical characteristics. Three individuals with CAG repeats of 37 (n = 1) and 38 (n = 2) were included in the current study. Although these individuals can be considered “affected”, they may have a lower risk of becoming symptomatic within their life span. Based on total motor scores (TMS), all patients were at the premanifest disease stage. Based on diagnostic confidence level scores (DCL), four patients presented some motor abnormalities, but none of them presented unequivocal motor signs of HD. Table 3 summarizes information about patients’ medication.

**Table 2:** Background clinical information of the patients’ cohort. CAG = cytosine, adenine, and guanine repeat size. DBS = disease burden score, a measure of proximity to clinical onset of the disease (Tabrizi et al., 2012), calculated as follows: DBS = age ×  (CAG‐35.5); the higher the DBS, the closer the patient’s proximity to disease onset. TMS = Total Motor Score out of 124 from the Unified Huntington’s Disease Rating Scale (UHDRS) Motor Diagnostic Confidence (Motor) – the higher the score stands for the higher the motor impairment. DCL = diagnostic confidence level, asks whether the participant “meets the operational definition of the unequivocal presence of an otherwise unexplained extrapyramidal movement disorder in a subject at risk for HD” (normal/no abnormalities = 0, non-specific motor abnormalities = 1, motor abnormalities that may be signs of HD = 2, motor abnormalities that are likely signs of HD = 3, motor abnormalities that are unequivocal signs of HD = 4).

CAG (SD, range)	Mean DBS (SD, range)	Mean TMS (SD, range)	Mean DCL (SD, range)
41.3 (2.14, 37–45)	236.15 (84.52, 80–450)	3.625 (5.11, 0–18)	0.875 (1.31, 0–3)

**Table 3:** Information about patients’ medication. Out of the 19 patients we assessed, 11 had been on stable medication for four weeks prior to taking part in the study.

Patient	Medication
1	Sumatriptan 10 g, Ventolin 400 mg, Mirtazapine 15 mg
2	Zolmitriptan, Loratadine
3	Ethinylestradiol 30mcg, Trimethoprim 400 mg
4	Ibuprofen 10 g, Paracetamol 10 g
5	Depomedrone + lidocaine 40 mg/ml
6	Paracetamol 10 g, Mebeverine 405 mg, Prochlorperazine 15 mg
7	Symbicort 10 g, Ventolin 10 g, Stemetil 10 g
8	Tamoxifen 20 mg, Venlafaxine 150 mg, Migraleve 10 g, Paracetamol 1000 mg, Bisoprolol 5 mg
9	Oxybutynin 1 mg, Cerazette 10 g, Amitriptyline 10 mg
10	Symbicort 10 g, Medroxyprogesterone 10 mg
11	Citalopram 30 mg, Aspirin 75 mg, Nasonex, Topamax 50 mg, Zopiclone 7.5 mg

### 2.2 MRI data acquisition and processing

• Imaging protocol

Complex multi-echo gradient-recalled echo (mGRE) data were acquired on a whole-body 7 T research MR-system (Siemens Healthcare GmbH, Erlangen, Germany) equipped with a 32-channel head receive/volume transmit coil (Nova Medical).

By using a prototype mGRE sequence, a single mid-sagittal 5 mm-thick slice was acquired, with in-plane field of view and resolution of 256 × 256 mm^2^ and 1 × 1 mm^2^, respectively. Acquiring a relatively thick slice afforded higher SNR and greater robustness in terms of slice misalignments across scans. The first echo time, echo spacing, and repetition time (TR) were set to TE1/ΔTE/TR = 1.62/1.23/100 ms, the flip-angle of the radiofrequency excitation pulse was 15˚ and a total of 25 bipolar gradient echoes were acquired. Specifically, we acquired this 20 times, with read gradient polarities inverted half way through, with a total acquisition time of 8 min and 32 s.

• Pre-processing steps

**Fig. 1:**
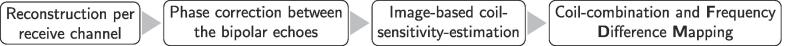
Schematic representation of the processing pipeline. FDM allowed removal of the RF-related phase offsets and linear effect of large-length-scale field perturbations, without perturbing the local non-mono-exponential WM signal.

The complex data were reconstructed per receive channel, followed by a complex multiplication of signals acquired with opposite read-gradient-polarities, in order to remove phase shift between adjacent echoes (Kleban et al., 2021). Image-based coil-sensitivity-estimation was then used to perform coil combination (Bydder et al., 2002, Roemer et al., 1990). Frequency difference maps (FDM) were calculated from the phase data to correct for the RF-related phase offsets and the effects of the non-local B_0_ field inhomogeneities (Kleban et al., 2021, Tendler and Bowtell, 2019). Finally, a 3rd degree spatial polynomial was fitted to the FDM data at each echo to correct for the residual eddy current effects. Fig. 1 summarises the pre-processing steps. Additional details on pre-processing can be found in the Appendix.

• Signal analysis

**Fig. 2:**
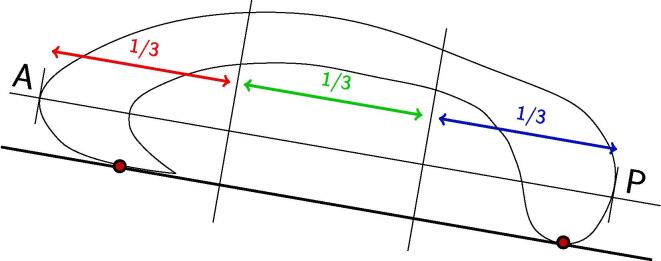
Schematic representation of the callosal segmentation protocol. The CC was segmented into three equal portions. Abbreviations: A = anterior; P = posterior.

For each scan we manually segmented the corpus callosum from a magnitude mGRE image acquired at TE = 15 ms and further parcellated it into anterior, middle and posterior portions as shown in Fig. 2. Magnitude and FDM data were averaged over each callosal segment.

**Fig. 3:**
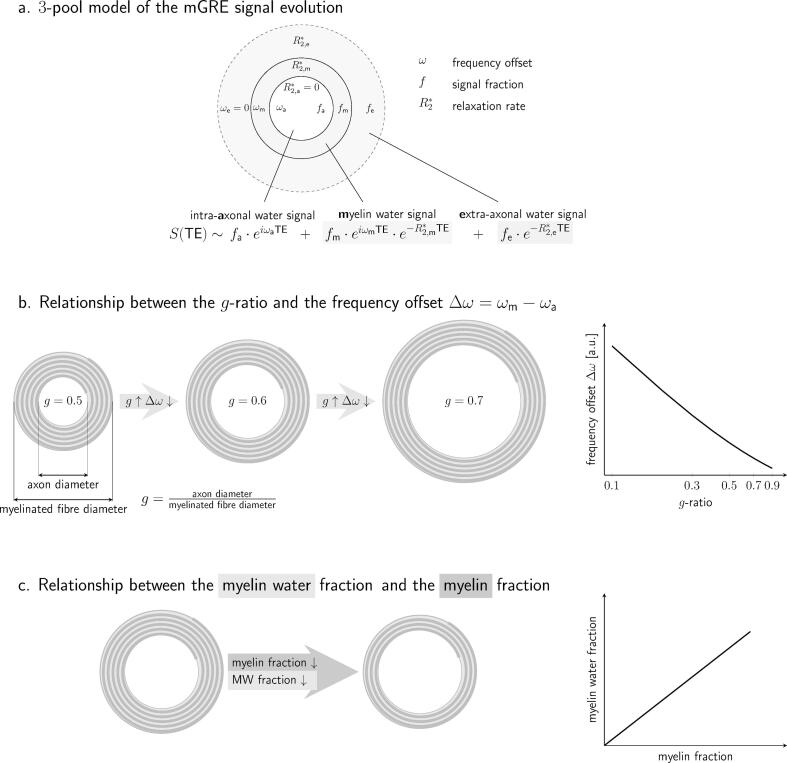
a. Schematic representation of the three-pool model for describing mGRE signal evolution in WM fibres perpendicular to B_0._ The signal is modelled as a superposition of complex myelin, intra- and extra-axonal water signals. b. Schematic representation of the relationship between g-ratio and the frequency offset between myelin and axonal water pools. An increase in g-ratio will be reflected by a decrease in Δω. c. Schematic representation of the relationship between the myelin water fraction and the myelin fraction. Such relationship highlights the potential of f_m_ as *in vivo* myelin marker.

FDM and magnitude signal evolution from each callosal segment were modelled using a three-pool-model of complex signal evolution (Fig. 3a), where myelin water, intra-axonal and extra-axonal compartments each have different signal amplitudes, decay rates, and frequency offsets (Cronin et al., 2017, Nam et al., 2015b, Nunes et al., 2017, Sati et al., 2013, Tendler and Bowtell, 2019, Thapaliya et al., 2018, Wharton and Bowtell, 2013):

(1) $$\begin{array}{c} S\left(t\right)=S_{\text{a}}\left(t\right)+S_{\text{e}}\left(t\right)+S_{\text{m}}\left(t\right)\sim f_{\text{a}}\cdot e^{\omega_{\text{a}}t}e^{-R_{2,\text{a}}^{*}t}+f_{\text{e}}\cdot e^{-R_{2,\text{e}}^{*}t}+f_{\text{m}}\cdot e^{\omega_{\text{m}}t}e^{-R_{2,\text{m}}^{*}t}.\# \end{array}$$

Here, a, e, and m denote intra-, extra-axonal and myelin water and the complex mGRE signal $S\left(t\right)$ is a superposition of their signals; f are the signal fractions, ω are the mean frequency offsets to the extra-axonal compartment, and $R_{2}^{*}$ are the transverse relaxation rates.

By including the frequency offset characteristics of the different water compartments (Sati et al., 2013, Wharton and Bowtell, 2012), this model offers reliable myelin water estimation (Nam et al., 2015b, Sati et al., 2013, van Gelderen et al., 2012). Furthermore, previous research has suggested that $\omega_{\text{m}}$ and $\omega_{\text{a}}$ may both depend on the g-ratio (Wharton and Bowtell, 2012). We represent the dependence of the frequency offset $\Delta \omega =\omega_{\text{m}}-\omega_{\text{a}}$ on the g-ratio in Fig. 3b, while the relationship between the myelin water signal and myelin content is shown in Fig. 3c.

$R_{2}^{*}$-values of the slow-decaying intra-axonal signal were constrained to 0 because of relatively low maximum TE (31 ms). This constraint helped to reduce the effect of the limited number of long TEs on the value estimation uncertainty at a cost of the potential under-/overestimation of the intra-/extra-axonal water signal fractions, respectively. Non-linear least-squares fitting was performed with initial parameter estimates and fitting boundaries based on previous literature (Sati et al., 2013, Tendler and Bowtell, 2019, Thapaliya et al., 2018, Wharton and Bowtell, 2012, Wharton and Bowtell, 2012) (Table 4).

**Table 4:** Parameter values (initial and range) used in non-linear least squares fitting. Choice of fitting boundaries was based on previous literature (Sati et al., 2013, Tendler and Bowtell, 2019; Thapaliya et al., 2017; Wharton and Bowtell, 2012, Wharton and Bowtell, 2013).

Parameter	f_m_	f_a_	f_e_	ω_a_/2π (Hz)	ω_m_/2π (Hz)	$R_{2,a}^{*}$(s^−1^)	$R_{2,m}^{*}$(s^−1^)	$R_{2,e}^{*}$(s^−1^)
Initial value	0.5	0.5	0.5	−8	30	0	150	25
Minimum value	0	0	0	−30	0	0	50	0
Maximum value	1	1	1	0	50	0	300	100

**Table 5:** Cognitive outcome variables employed to assess patient-control differences in cognition. A short description of the task is provided, together with a list of outcome variables and cognitive domains assessed.

Task	Description	Outcome variable	Cognitive process assessed
N-back (Kirchner, 1958**)**	For each letter presented in a sequence, subjects judge whether it matches the one presented ‘n’ items ago.	Percentage of correct responses in the 1) 1-back and 2) 2-back condition.	Encoding, temporary storage and updating of stored information with new upcoming information, inhibition of irrelevant items (Ecker et al., 2014, Rey-Mermet et al., 2018).
Digit span test from the WAIS-R (Wechsler, 1997**)**	Participants have to repeat a sequence of digits in the same order in which they were presented. The number of digits in each sequence is sequentially increased.	Maximum span of digits recalled.	Verbal working-memory capacity.
Visual patterns test (Della Sala et al., 1997**)**	Participants are shown a checkerboard-like grid, with the squares in the grid each randomly coloured. They are then shown a blank grid and are asked to reproduce each grid. The size of the grid is sequentially increased.	Maximum grid size recalled correctly.	Spatial working-memory capacity.
Eyes test (Baron‐Cohen et al., 2001)	36 still pictures of the eye regions within faces expressing different emotional states are presented. Subjects are asked to match a list of provided emotional tags to the emotions displayed in the pictures.	Number of emotional states correctly matched.	Social cognition and mentalising.
Finger tapping task (Freeman, 1940)	Participants are required to press a button on the keyboard as quickly as possible with the index finger of their dominant hand.	Mean number of taps across 3 trials.	Motor speed.

Pre-processing and signal analysis were performed in Matlab (Matlab, The Mathworks, Natick, MA).

### 2.3 Cognitive tests

Cognitive performance was assessed in premanifest HD patients and age, sex and education matched healthy controls in the following tests: (1) the n-back task (Kirchner, 1958); (2) the digit span test from the Wechsler Adult Intelligence Scale-Revised (WAIS-R) (Wechsler, 1997); (3) the visual patterns test (Della Sala et al., 1997); (4) the Reading the Mind in the Eyes test (Baron‐Cohen et al., 2001), hereafter referred to as the eyes test; and (5) the finger tapping task (Freeman, 1940). Tests (3) and (4) were administered as paper and pencil tests, tests (1), (2), (5), and (6) by using the computerized version provided by the Psychology Experiment Building Language (PEBL) Test Battery (Mueller and Piper, 2014). Overall, we obtained a total of 6 cognitive outcome measures, which are summarised in Table 5, together with a short description of each task.

### 2.4 Statistical analysis

We selected $f_{\text{m}}$, $\omega_{\text{m}}$, $\omega_{\text{a}}$, and $\Delta \omega =\omega_{\text{m}}-\omega_{\text{a}}$ obtained from mGRE complex signal analysis to perform further statistical analyses, as these metrics may reflect myelin changes in WM (see diagrams in Fig. 3bc).

• Reproducibility study

To assess the test–retest reproducibility of the data, we obtained the Fréchet distance (Fréchet, 1957) between FDM curves to measure their similarity. This method takes into account the location and ordering of points along the curves. Specifically, given two curves, Q and P, the Fréchet distance is defined as the minimum cord-length sufficient to join a point traveling forward along P and one traveling forward along Q. Furthermore, the coefficients of variation (CVs, the ratio of the standard deviation to the mean) across the 5 visits were computed for f_m,_ ω_a,m_, and Δω for each participant, for each segment. Finally, we used the R package cvequality (Version 0.1.3, Marwick and Krishnamoorthy 2019) to compute the ‘modified signed-likelihood ratio test for equality of CVs’ (Krishnamoorthy and Lee, 2014). This allowed us to test for significant differences between the CVs across the three segments, for each metric.

• HD study

As greater measurement reproducibility was detected in the posterior segment of the CC, this was chosen as the region of interest for the assessment of patient-control differences in callosal myelin content. Age, but not TOPF-UK IQ, was found to be significantly correlated with both f_m_ and Δω. Hence age was included as a covariate in the analysis of group effects. Specifically, multiple regression analyses, assessing the effect of group, age, and a group-by-age interaction on f_m_ and Δω, were run in order to assess whether these metrics could disentangle age-related changes from pathologic HD-associated neurodegeneration. We performed regression diagnostics and examined QQ plots and outlier profiles to detect any values above or below the upper/lower boundary of 95% confidence intervals of the slope of the regression line. As a sanity check, we also confirmed our results by running robust linear regression analyses, using the *lmRob* R function from the robust package (Wang et al., 2009), which handles small sample sizes, skewed distributions and outliers (Wilkinson, 1999).

Principal component analysis (PCA) was employed to reduce the complexity of the cognitive data and hence the problem of multiple comparisons, as well as to increase experimental power. We examined potential confounding effects of age or TOPF-UK IQ on the extracted components. Age was included as a covariate in the model, which explored the effect of group, age, and a group-by-age interaction on the scores on the extracted components. We interpreted any significant effect by referring to the variables with significant component loadings, as highlighted in bold in Table 7.

As a significant group effect was detected on f_m,_ Spearman’s rho correlation coefficients were calculated in patients between this metric, DBS, and scores on the first cognitive component (i.e., the component capturing the greatest amount of variability in the data), to assess disease-related brain-function relationships. We corrected for multiple comparisons with the Bonferroni correction with a family-wise alpha level of 5% (two-tailed). Significant correlations were further assessed with partial correlations to control for age as potentially mediating variable.

All statistical analyses were carried out in R Statistical Software (Foundation for Statistical Computing, Vienna, Austria) and Matlab (Matlab, The Mathworks, Natick, MA).

## 3 Results

### 3.1 The precision of FDM in the corpus callosum is anatomically variable

**Fig. 4:**
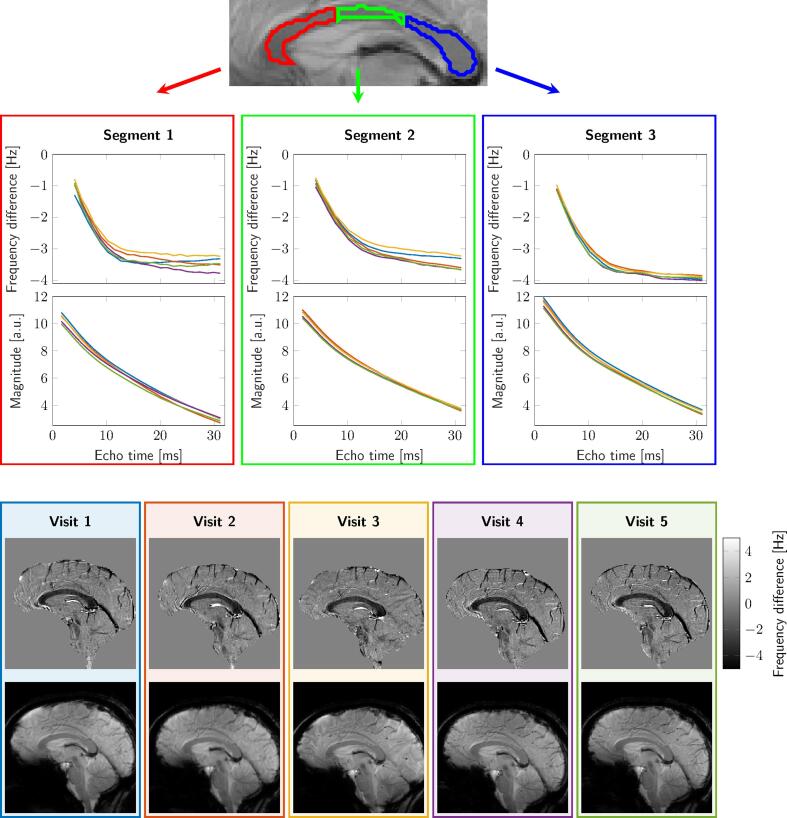
Example reproducibility data from one representative subject. The top image shows the callosal parcellation overlaid on a magnitude image; the plots show frequency difference and magnitude of signal as function of TE for anterior/middle/posterior callosal segments; corresponding frequency difference and magnitude images at TE = 15 ms for five visits are shown at the bottom.

Fig. 4 shows an example of FDM and magnitude signal evolution as a function of TE, for anterior/middle/posterior callosal segments, and the corresponding images at TE = 15 ms for five visits.

**Fig. 5:**
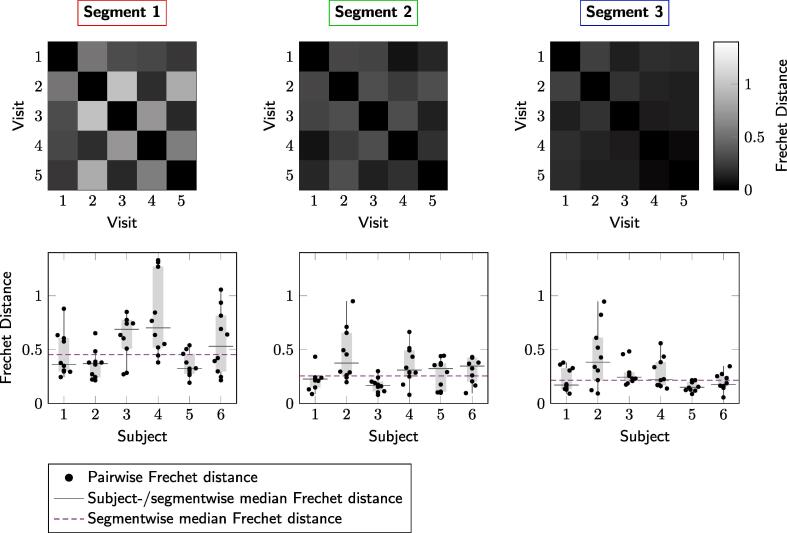
Assessment of the similarity of frequency difference curves. Top: Fréchet distance matrices from a representative participant for the three callosal segments. Bottom: Repeatability of frequency difference evolution across five visits for all subjects.

**Fig. 6:**
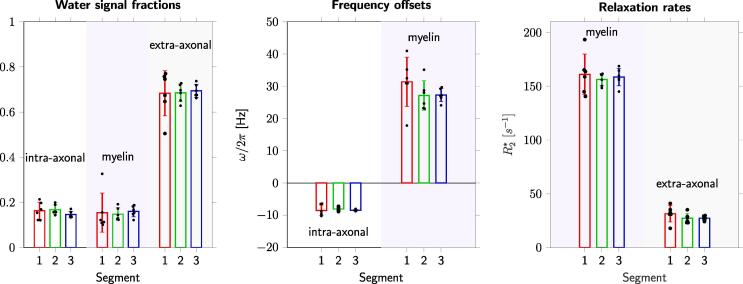
Fitting parameters estimated from the three-pool model, grouped for all subjects. Colours represent the three callosal segments (red/green/blue for anterior/mid/posterior segments, respectively). The first plot shows signal fractions of myelin, intra-/extra-axonal water; the frequency offsets are displayed in the middle plot; the last plot shows the relation rates of myelin and extra-axonal water signals. (For interpretation of the references to colour in this figure legend, the reader is referred to the web version of this article.)

Analysis of curve similarity (Fig. 5) and fitting parameter repeatability (Fig. 6, Table 6) suggested greater reproducibility of measures in the posterior callosal portion as compared to the anterior sections. This is shown by generally lower Fréchet distance values and by overall low coefficients of variation calculated for the fitting parameters, which ranged between 3.72% and 12.02%, in this region (Table 6). The modified signed-likelihood ratio test for equality of CVs confirmed that CVs in the posterior callosal segment were significantly smaller across all measures [f_m_: p = 0.02; ω_a_/2π (Hz): p < 0.001; ω_m_/2π (Hz): p < 0.001; Δω//2π) (Hz): p = 0.020].

**Table 6:** Reproducibility of myelin water signal fraction (f_m_), frequency offsets of axonal (ω_a_/2π) and myelin (ω_m_/2π) water pools, and difference in frequency offsets between myelin and axonal water pools (Δω/2π)). Means, standard deviation (SD) and coefficients of variation (CV) are reported for each value, across the different segments.

Metric	Callosal segment	Mean	SD	CV (%)
f_m_	Segment 1	0.15	0.08	49.14
ω_a_/2π (Hz)		−8.5	2.0	24.84
ω_m_/2π (Hz)		31.4	10.2	37.01
Δω/2π (Hz)		39.9	7.95	23.02
f_m_	Segment 2	0.15	0.04	26.98
ω_a_/2π (Hz)		−7.9	1.1	13.42
ω_m_/2π (Hz)		27.1	4.4	16.60
Δω/2π (Hz)		35.2	3.70	11.00
f_m_	Segment 3	0.16	0.02	12.02
ω_a_/2π (Hz)		−8.5	0.6	7.07
ω_m_/2π (Hz)		27.3	2.1	7.61
Δω/2π (Hz)		35.8	1.80	5.10

### 3.2 Multi-compartment analysis reveals HD-related myelin changes in the posterior segment of the CC

**Fig. 7:**
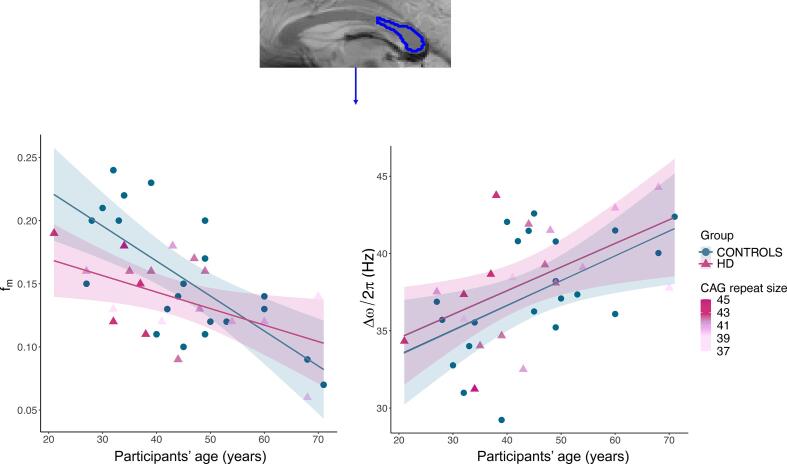
Top: Parcellation of segment 3 of the CC overlaid on a magnitude image; the same protocol as the one detailed in the reproducibility study section was utilised. Left: Regression plot showing the relationship between age and f_m_, split by group. Both age and group were significant predictors of variance in f_m._ No significant interaction effect was detected. Right: Regression plot showing the relationship between age and Δω, split by group. Age was a significant predictor in the model, while group did not significantly predict variance in this metric. No significant interaction effect was detected. HD data points are coloured by CAG repeat size: older HD carriers presented shorter CAG repeat mutation and a trend for a greater overlap in f_m_ with values of age-matched healthy controls, indicating that CAG repeat size may directly affect myelin content in premanifest HD.

Fig. 7 plots the relationship between age and f_m_, and between age and Δω, split by group, in Segment 3 of the CC. Group and age explained 45% of the variance in f_m_ [R^2^ = 0.45, F(4, 35) = 7.18, p < 0.001]. Specifically, it was found that group significantly predicted f_m_ values in this portion of the CC (β = -0.13, p = 0.03), as did age (β = -0.82, p < 0.001). However, we did not detect a significant group-by-age interaction effect (β = 0.82, p = 0.08) (Fig. 7, left). Overall, HD patients presented a flatter age-related variation in this metric, with values being overall lower, especially in younger subjects.

On the other hand, age and group explained 31% of the variance in Δω [R^2^ = 0.31, F(4, 35) = 3.87, p = 0.01]. Age was found to be a significant predictor of variance in this metric (β = 0.55, p = 0.009), so that being older was associated with a greater Δω in this sample. However, belonging to the patient or the control group did not have a significant effect on this measure (β = 0.13, p = 0.8) (Fig. 7, right).

### 3.3 Premanifest HD is associated with greater age-related decline in executive functions

**Fig. 8:**
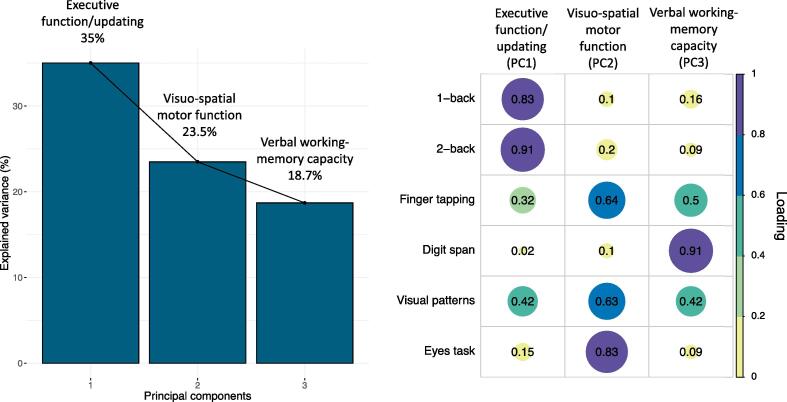
PCA of the cognitive data with varimax rotation. Left: PCA scree plot. Right: Plot summarising how each variable is accounted for in every principal component. The absolute correlation coefficient is plotted. Colour intensity and the size of the circles are proportional to the loading. Three components explaining over 77% of the data variability were extracted. PC1 loaded on n-back task performance and was therefore summarized as “executive function” component; PC2 was summarized as “visuo-spatial motor function” component; PC3 loaded on digit span task performance and was therefore summarized as “working memory” component. 7 control cases did not complete all tests and were therefore excluded from the PCA. The final sample size for the PCA was n = 19 for the HD group and n = 14 for the control group.

With PCA of cognitive test scores, the first three components explained 77.7% of the variability in performance in the administered tests (Fig. 8). The first component loaded positively on variables from the n-back task and was therefore summarized as an “executive function/updating” component. Principal component 2 (PC2) was summarised as a “visuo-spatial motor function” component. Finally, PC3 was summarized as a “verbal working memory capacity” component as this loaded mostly on the digit span task.

**Fig. 9:**
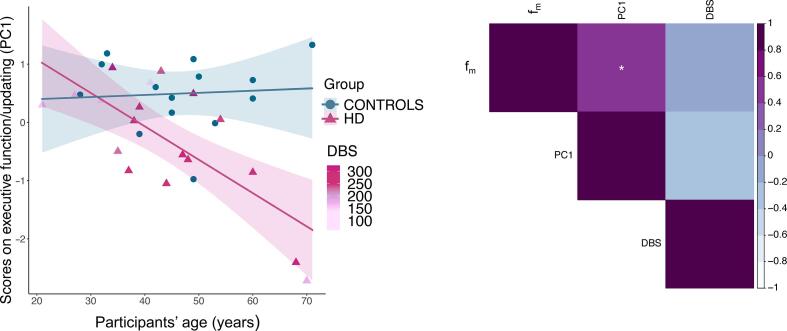
Left: Relationship between executive function scores and age, in patients and controls. A significant interaction effect between group and age was detected, suggesting that the group difference in executive function scores is larger at later ages. HD data points are coloured by DBS. Older HD patients tend to be closer to disease onset, possibly confounding the effect of age on this measure. Right: Relationship between f_m_ executive function scores and DBS in patients, Bonferroni-corrected. A significant positive correlation was found between f_m_ and executive function scores. Colour intensity is proportional to the strength and direction of the correlation. * p < 0.05, ** p < 0.01, *** p < 0.001, Bonferroni-corrected.

We detected no significant main effect of group (although a trend was present) (β = 1.9, p = 0.059) or age (β = 0.004, p = 0.815) on the executive function/updating component, but a significant interaction effect was present between group and age [β = −0.06, p = 0.006, R^2^ = 0.52, F(3,28) = 10.6, p = 0.006], indicating that while younger HD patients present executive function scores which tend to overlap with those of healthy controls, the gap in performance between the two groups is significantly larger at later ages (Fig. 9, left).

On the other hand, the main effects of group (β = −1.71, p = 0.23) and age (β = −0.036, p = 0.14), and the interaction effect between group and age (β = 0.03, p = 0.31) on the visuo-spatial motor function component were not significant [R^2^ = 0.09, F(3,28) = 0.94, p = 0.43]. Similarly, we did not detect a significant main effect of group (β = 0.03, p = 0.82) and age (β = 0.03, p = 0.15), nor a significant interaction effect between group and age (β = −0.003, p = 0.91) on the verbal working memory capacity component [R^2^ = 0.14, F(3,28) = 1.63, p = 0.20].

### 3.4 Interindividual differences in myelin content in the posterior callosum relate to executive function but not to proximity to disease onset in premanifest HD patients

We detected a significant positive correlation between the patients’ inter-individual variation in f_m_ and their scores on the executive component (r = 0.542, p = 0.02, corrected p = 0.04) (Fig. 9, right); although a positive trend remained, this relationship was no longer significant after partialling out age (r = 0.37, p = 0.13). Additionally, there was no association between inter-individual variation in f_m_ and proximity to disease onset as measured with DBS (r = −0.09, p = 0.686, corrected p = 1).

## 4 Discussion

### 4.1 Reproducibility of the mGRE signal across the CC

The reproducibility of FDM evolution and 3-pool model derivatives in the CC was found vary according to anatomical location, with more precise fitting parameters in the posterior portion of the callosum. Lower reproducibility of the data in the anterior portions of the CC could be attributed to in-flow artifacts from the anterior cerebral artery (Nam et al., 2015a, Tendler and Bowtell, 2019). Potential solutions and their limitations have been discussed in previous work, such as the application of flow saturation RF pulses to the inferior portion of the head (Nam et al., 2015a).

The estimated fitting parameters for the intra-axonal and myelin water frequency offsets (−7.9 to −8.5 Hz and 27.1 to 37.4 Hz, respectively) and the myelin water signal fraction (0.15 to 0.16) were consistent with previously reported values (Sati et al., 2013, Tendler and Bowtell, 2019, Thapaliya et al., 2018, Wharton and Bowtell, 2012). On the other hand, the relative signal fraction between intra- and extra-axonal compartments was lower than in other studies (Tendler and Bowtell, 2019). This could be attributed to the constraint we placed on the intra-axonal water $R_{2}^{*}$ when modelling the data, which we introduced in order to reduce the effect of the limited number of long TEs on the value estimation uncertainty. Please refer to Appendix B for simulations we conducted on the impact of setting $R_{2,\text{a}}^{*}=0$ on MW signal fraction values. Finally, slice positioning relative to the CC, and the subsequent ROI segmentation, may also have introduced some variability in test–retest scans signal and estimated parameters.

### 4.2 Application of the 3-pool model to assess myelin content in premanifest HD

We observed significantly lower f_m_ values in premanifest HD patients compared to healthy controls, suggesting the presence of myelin impairment (Bartzokis et al., 2007). Previous animal studies have already shown a link between HD pathology and changes in myelin-associated biological processes at the cellular and molecular level (Ferrari Bardile et al., 2019, Huang et al., 2015, Jin et al., 2015, Teo et al., 2016). Crucially, however, previous *in vivo* investigations of WM changes in HD have predominantly employed indices from DT-MRI (Pierpaoli & Basser, 1996) or magnetization transfer ratio (MTR) imaging (Henkelman et al., 2001). These indices may be influenced by a multitude of processes affecting tissue microstructure and biochemistry (Beaulieu, 2002, De Santis et al., 2014, Harsan et al., 2006, Henkelman et al., 1993, Wheeler-Kingshott and Cercignani, 2009). In contrast, our study exploited FDM and a three-pool modelling of the mGRE signal to afford improved WM compartmental specificity, and to estimate f_m_ as a marker of myelin content. Importantly, histological evidence shows that this metric is less sensitive to concomitant pathological processes such as inflammation (Gareau et al., 2000), suggesting that this may be a more specific measure of tissue myelination than other MRI-derived measures. Our results highlight the potential of f_m_ in helping to better understand HD pathogenesis and progression, and to gain further insight into the biological basis of WM microstructural changes in the HD brain. Additionally, they suggest the presence of myelin breakdown as an early feature of HD progression and are consistent with evidence from a quantitative magnetisation transfer study (Bourbon-Teles et al., 2019), which demonstrated reductions in the macromolecular proton fraction - a myelin sensitive measure - in HD patients.

In this study, we did not detect a group effect on Δω, while this parameter was shown to significantly increase with age. Based on the model proposed by Wharton and Bowtell (2012), this might reflect two processes: i. the g-ratio decreases with age; ii. the magnetic susceptibility difference between the myelin sheath and the extra-axonal pool increases with age. The first suggestion contradicts previous studies showing g-ratio increases with age (Peters, 2009). However, as fibres with smaller diameters tend to have slightly lower g-ratios (Berthold et al., 1983), a selective loss of large-diameter axons would lead to an overall reduced voxel-averaged g-ratio (Cercignani et al., 2017). This scenario is a plausible explanation of our finding, as fibres in the posterior portion of the CC are predominantly large and early myelinated (Aboitiz et al., 1992). Additionally, an increase in iron-containing glial cells in the surrounding extra-axonal space could result in the increase of the susceptibility difference between myelin sheath and the extra-axonal space (Xu et al., 2015). This is consistent with evidence showing that over the course of aging, iron accumulates in the brain (Connor et al., 1990, Dexter et al., 1991, Jellinger et al., 1990, Zecca et al., 2004).

With regards to the cognitive assessments, our results indicate that executive functions, and specifically the updating of relevant information, tend to deteriorate to a larger extent with age in HD patients, compared to controls. Nevertheless, these results might have been confounded by stage of disease progression, as older participants presented a higher DBS. Understanding of the nature of cognitive deficits associated with HD pathogenesis and progression provide useful guidance for future research into the efficacy of cognitive training and rehabilitation approaches in HD (Andrews et al., 2015). Our findings suggest that such approaches might be more effective early in the lifetime and in disease progression.

In the present study, we also found that patients’ inter-individual variability in f_m_ was positively associated with their scores on the executive/updating component. Although anterior, rather than posterior, callosal portions have been normally associated with frontal‐lobe‐mediated executive functions (e.g. Jokinen et al., 2007), posterior callosal fibres are connected to posterior parietal areas of the brain (Goldstein et al., 2020); these areas have been associated with top-down modulation during inhibition and attention processes (Erickson et al., 2009, Hopfinger et al., 2000), which are recruited during maintenance and updating of relevant information. It is therefore plausible that microstructural variation in this callosal segment may impact performance in this cognitive domain. Additionally, although a positive trend remained, this relationship was no longer significant after partialling out age, stressing the important role of aging in both myelin content in the brain, and executive functioning (Grieve et al., 2007, Guttmann et al., 1998, Lintl and Braak, 1983, Pakkenberg et al., 2003). Interestingly, we found no association between inter-individual variation in f_m_ and proximity to disease onset as measured with DBS. This suggests that myelin differences may precede the onset of clinical symptoms in HD and may not directly relate to disease stages.

### 4.3 Challenges to the interpretation of mGRE signal evolution

In this work, the complex WM mGRE signal was modelled as a superposition of three water signals which were assigned to the MW, intra-, and extra-axonal pools. Similar to previous work, the pool with the shortest $T_{2}^{*}$ and the largest (positive) frequency offset was assigned to the myelin water (MW) pool. This representation assumes that chemical exchange between the MW pool and the intra-/extra-axonal water pools is negligibly small and that the myelin sheath is the dominant source of magnetic susceptibility effects in WM.

However, it has previously been suggested that magnetisation exchange can affect the interpretation of tissue compartmentalisation from MR signals or NMR frequency in brain tissues (Shmueli et al., 2011, van Gelderen and Duyn, 2019). Other work has suggested that the presence of iron-rich oligodendrocytes and the geometry of axons may have an impact on compartmental frequency distributions and consequently on the MR signal (Xu et al., 2015, Xu et al., 2018). Additionally, a previous study performed MWI using $T_{2}$-weighted techniques on a *post-mortem* brain before and after iron extraction by reductive dissolution and reported a 26% reduction in the MW signal fraction (Birkl et al., 2019). Based on the reportedly excellent agreement between mGRE and $T_{2}$-weighted MWI techniques at 3 T (Alonso-Ortiz et al., 2018b), it is plausible that iron deposition may have altered the MW signal fraction in our mGRE experiments at 7 T. Finally, the similarity between intra- and extra-axonal mGRE signal evolutions may further confound the accuracy of compartmentalisation, especially at lower magnetic fields and short echo times (Chan and Marques, 2020). Crucially, this work exploited ultra-high magnetic fields and this may have allowed a better separation between the intra- and extra-axonal signals (Alonso-Ortiz et al., 2018a). Nevertheless, simultaneous variation of multiple experimental parameters and additional information derived from other MRI contrasts may further improve mGRE signal interpretation (Chan and Marques, 2020, Kleban et al., 2020).

## 5 Conclusions and future directions

In summary the present study exploited, for the first time in HD research, the sensitivity of the 3-pool model analysis of the complex mGRE signal to quantify myelin changes in premanifest HD. Results stress the potential of this marker in helping to better understand HD pathogenesis and progression, and provide original in-vivo evidence for reductions in f_m_, a proxy MRI marker of myelin, in human premanifest HD. Expanding on evidence from pathology and animal studies, our results suggest that myelin breakdown is an early feature of HD progression, and lend supporting evidence to the progression model suggesting that early myelinated fibres are affected by myelin breakdown early in the disease (Bartzokis et al., 2007).

Our findings were based on a relatively small sample size and warrant replication in larger samples. In addition, three individuals with CAG repeats of 37 and 38 were included in the current study. Though these individuals can be considered “affected”, they may have a lower risk of becoming symptomatic within their life span, and some studies have chosen to exclude individuals with reduced disease penetrance (e.g. Rubinsztein et al., 1996). Although the aim of this study was to look at the premanifest, rather than symptomatic, stage of the disease, their inclusion in the current study may raise the possibility of Type II errors when generalizing to the wider population of individuals with HD. Though this did not seem to be the case in the present study as we did detect a group effect, future studies may want to replicate these results in a sample of premanifest HD patients with full disease penetrance. Finally, while it is tempting to assign, unequivocally, a one-to-one correlation between changes in MRI signal and biological properties, and thus interpret these changes purely in terms of changes in myelination, these findings need to be interpreted with caution. For example, changes in iron content cannot be ruled out.

Future studies should assess HD-related changes in f_m_ longitudinally rather than cross-sectionally and investigate how these changes relate to clinical symptoms over time, to further understand the utility of this metric as a marker of early disease development and progression. Additionally, this study utilised a single-slice technique, and investigated a small portion of the CC, thus limiting the assessment of global diffuse tissue damage. Of special interest for future investigations might be to increase brain coverage and assess how f_m_ changes may differentially impact early and later myelinating regions in the premanifest HD brain. With regards to the observed increase of Δω with age, MR axon radius mapping using diffusion MRI and ultra-strong gradients (Veraart et al., 2020, Veraart et al., 2021) may help elucidate whether a selective loss of large-diameter axons is producing this effect.

*Ethics approval and consent to participate*: The study was approved by the Cardiff University School of Psychology Ethics Committee and by the local National Health Service (NHS) Research Ethics Committee (Wales REC 5 18/WA/0172) and all participants provided written informed consent.

## CRediT authorship contribution statement

**Chiara Casella:** Conceptualization, Methodology, Validation, Formal analysis, Investigation, Data curation, Writing - original draft, Visualization, Project administration, Funding acquisition. **Elena Kleban:** Conceptualization, Methodology, Software, Validation, Formal analysis, Investigation, Resources, Writing - review & editing, Visualization, Supervision, Project administration. **Anne E. Rosser:** Resources. **Elizabeth Coulthard:** Resources. **Hugh Rickards:** Resources. **Fabrizio Fasano:** Software. **Claudia Metzler-Baddeley:** Conceptualization, Writing - review & editing, Supervision, Project administration. **Derek K. Jones:** Conceptualization, Methodology, Writing - review & editing, Supervision, Project administration, Funding acquisition.

## Declaration of Competing Interest

The authors declare that they have no known competing financial interests or personal relationships that could have appeared to influence the work reported in this paper.

## Figures and Tables

**Table B1:** Input parameters for the simulated signal. The MW signal fraction f_m_ was varied from 0.1 to 0.2 for Fig. B1, and the intra-axonal relaxation rate was varied from 15 to 35 s^−1^ for Fig. B2.

$\omega_{\text{a}}$[Hz]	$\omega_{m}$[Hz]	$R_{2,m}^{*}$[s^−1^]	$R_{2,\text{a}}^{*}$[s^−1^]	$R_{2,e}^{*}$[s^−1^]	$f_{\text{a}}/f_{\text{e}}$	$f_{\text{m}}$
−6	30	160	25	40	2/3	0.15

**Fig. B1:**
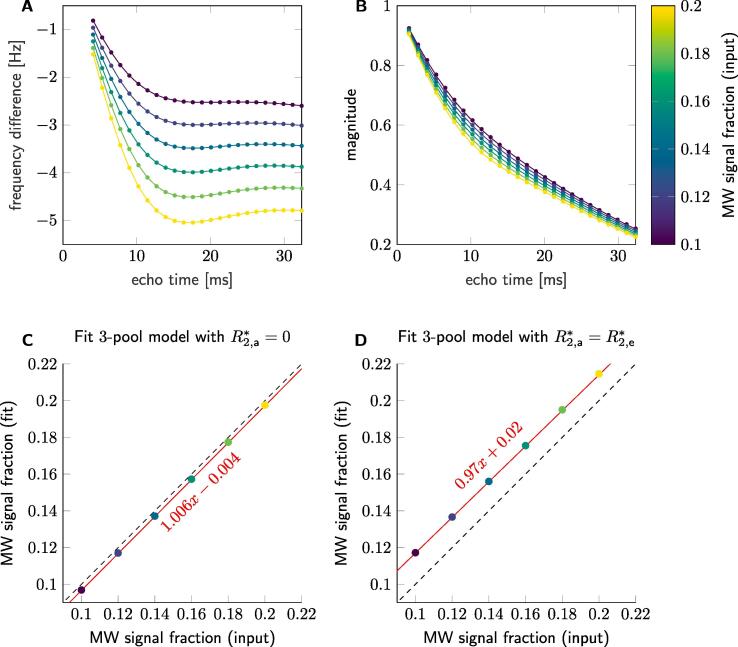
Frequency difference (A) and magnitude (B) were estimated from a complex signal, which was simulated for myelin water signal fraction, $f_{\text{m}}$, ranging from 0.1 to 0.2. Other parameters used to simulate the 3-pool complex signal are listed in Table 1. The colour bar corresponds to $f_{\text{m}}$. The 3-pool model was then fitted for two assumptions: $R_{2,\text{a}}^{*}=0$ and $R_{2,\text{a}}^{*}=R_{2,\text{e}}^{*}$, and the estimated MW signal fraction values were plotted against the input values in (C) and (D), respectively. The red line represents the linear relationship between the estimated f_m_ and the input f_m_, slopes and intercepts are indicated in the plots.

**Fig. B2:**
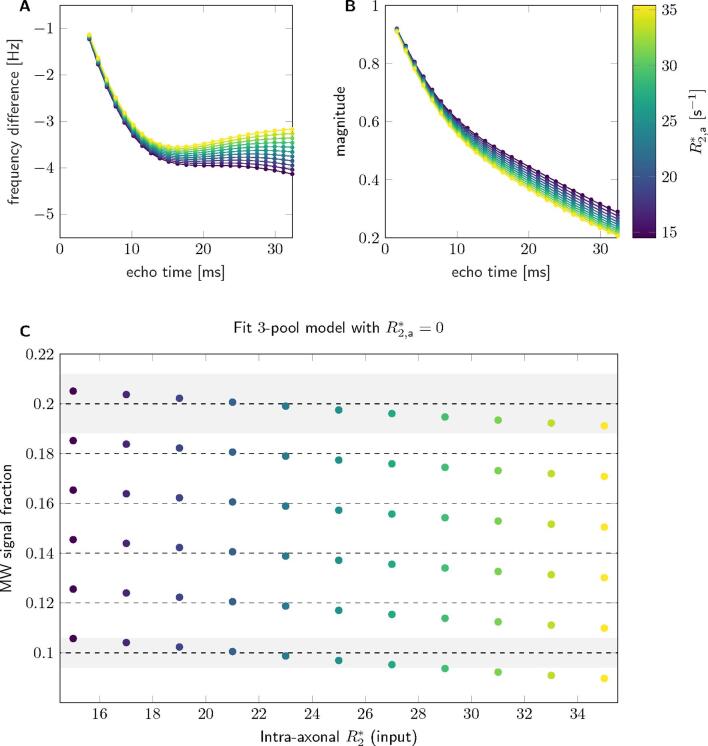
Frequency difference (A) and magnitude (B) were estimated from a complex signal, which was simulated for intra-axonal relaxation rate $R_{2,\text{a}}^{*}$ ranging from 15 to 35 s^−1^. Other parameters used to simulate the 3-pool complex signal are listed in Table 1. In C, MW signal fraction was estimated under assumption $R_{2,\text{a}}^{*}=0$ and plotted against the input intra-axonal relaxation rate. Dashed lines indicate the input MW signal fractions. Gra areas are equivalent to 12% (±6%) of 0.1 and 0.2, respectively. The value of 12% is equivalent to coefficient of variation of $f_{\text{m}}$ in the splenium of the corpus callosum estimated in the reproducibility study (cf., Table 6). The colour bar corresponds to $R_{2,\text{a}}^{*}$.

## Appendix A: Details on mGRE signal pre-processing

In this work the mGRE signal was acquired using bipolar gradient readouts with the polarity of the first echo inverted halfway through the number of repeats. We use ± sign to indicate where the polarity of the read gradient may affect the corresponding mGRE signal.

The complex mGRE signal was reconstructed per receive channel k and gradient echo n at corresponding echo time ${\text{TE}}_{n}$ and can be represented as follows (Sati et al., 2013, Tendler and Bowtell, 2019, Thapaliya et al., 2018, Wharton and Bowtell, 2012, Nam et al., 2015a, Nam et al., 2015b, Kleban et al., 2021):

(A.1) $$\begin{array}{c} S_{n,k}^{\pm }\left(\vec{r},TE_{n}\right)\sim S_{0}\left(\vec{r}\right)\cdot e^{i\Omega \left(\vec{r}\right)TE_{n}}\cdot e^{i\phi_{0,k}\left(\vec{r}\right)}\cdot e^{\pm {\left(-1\right)}^{n}i\psi_{n}}\cdot F\left(\vec{r},TE_{n}\right) \end{array}$$

$F_{n}=F\left(\vec{r},{\text{TE}}_{n}\right)$ represents the complex WM signal as a function of echo time, which in this work was modelled using a three-pool model.

The exponentials preceding $F_{n}$ in Eq. (A.1) reflect macroscopic spatio-temporal phase effects which contribute to the total complex mGRE signal and need to be addressed before $F_{n}$ can be analysed (Lee et al., 2018, Sati et al., 2013, Tendler and Bowtell, 2019, Thapaliya et al., 2018, Wharton and Bowtell, 2013):

1) The frequency Ω represents the effect of large-length-scale field perturbations; 2) The phase $\phi_{0,k}$ describes collective phase offsets including those specific for each receiver channel; 3) The phase $\psi_{n}$ highlights phase differences arising when signal from the same echo is acquired with opposite gradient polarities.

We correct for the phase effects in following steps:

The phase shifts $\psi_{n}$ can be removed by multiplying $S_{n,k}^{+}$ by $S_{n,k}^{-}$ (Kleban et al. 2021):

(A.2) $$\begin{array}{c} S_{n,k}^{+}\cdot S_{n,k}^{-}\sim S_{0,k}{\left(\vec{r}\right)}^{2}\cdot e^{i2\Omega TE_{n}}\cdot e^{i2\phi_{0,k}}\cdot F_{n}^{2} \end{array}$$

Remove phase offsets, particularly those varying between channels (Tendler and Bowtell, 2019, Kleban et al., 2021, Bydder et al., 2002):

(A.3) $$\begin{array}{c} {\left[S_{n,k}^{+}\cdot S_{n,k}^{-}\right]}^{\prime }=\frac{S_{n,k}^{+}\cdot S_{n,k}^{-}}{S_{1,k}^{+}\cdot S_{1,k}^{-}}\cdot \left|S_{1,k}^{+}\cdot S_{1,k}^{-}\right|\sim S_{0,k}{\left(\vec{r}\right)}^{2}e^{i2\Omega \left(TE_{n}\right)}\cdot \frac{F_{n}^{2}}{F_{1}^{2}} \end{array}$$

This is the first step of frequency difference mapping which in this case also removes phase inconsistencies between the receive channels, so that the coil combination can be applied next.

The signals from all receive channels are combined by calculating the sum of over the channels weighted by the channels’ sensitivity $m_{k}$ (Bydder et al., 2002, Roemer et al., 1990).

(A.4) $$\begin{array}{c} M_{n}=\frac{1}{\sum_{k}m_{k}^{2}}\sum_{k}\sqrt{\left|{\left[S_{n,k}^{+}\cdot S_{n,k}^{-}\right]}^{\text{'}}\right|}\cdot m_{k}, \end{array}$$

(A.5) $$\begin{array}{c} S_{n}^{\text{'}}=\frac{1}{\sum_{k}m_{k}^{2}}\sum_{k}e^{i\arg\left({\left[S_{n,k}^{+}\cdot S_{n,k}^{-}\right]}^{\text{'}}\right)}\cdot m_{k}\sim e^{i2\Omega \left({\text{TE}}_{n}\right)}\cdot \frac{F_{n}^{2}}{F_{1}^{2}}, \end{array}$$

where $m_{k}$ was approximated from signal magnitude of the first echo:

(A.6) $$\begin{array}{c} m_{k}=\frac{\sqrt{\left|{\left[S_{1,k}^{+}\cdot S_{1,k}^{-}\right]}^{\text{'}}\right|}}{\sqrt{\sum_{l}\left|{\left[S_{1,l}^{+}\cdot S_{1,l}^{-}\right]}^{\text{'}}\right|}} \end{array}$$

The final step of FDM calculation aims to remove the effects of Ω (Tendler and Bowtell, 2019, Kleban et al., 2021):

(A.7) $$\begin{array}{c} S_{n}^{\text{'}\text{'}}=\frac{S_{n}^{\text{'}}}{{\left[S_{2}^{\text{'}}\right]}^{n-1}}={\left[\frac{F_{n}\cdot {\left[F_{1}\right]}^{n-2}}{{\left[F_{2}\right]}^{n-1}}\right]}^{2}, \end{array}$$

which can be translated to frequency:

(A.8) $$\begin{array}{c} {\text{FDM}}_{n}=\frac{\arg\left(S_{n}^{\text{'}\text{'}}\right)}{4\pi \left[n-2\right]\Delta \text{TE}} \end{array}$$

As evident from Eq. (A.7), each echo contributes to the estimation of the frequency difference.

## Appendix B: Simulations to address the impact of $R_{2,\text{a}}^{*}=0$ assumption on MW signal fraction

We simulated the complex mGRE signal using a 3-pool model:

(B.1) $$\begin{array}{c} S\left(t\right)=S_{\text{a}}\left(t\right)+S_{\text{e}}\left(t\right)+S_{\text{m}}\left(t\right)\sim f_{\text{a}}\cdot e^{\omega_{\text{a}}t}e^{-R_{2,\text{a}}^{*}t}+f_{\text{e}}\cdot e^{-R_{2,\text{e}}^{*}t}+f_{\text{m}}\cdot e^{\omega_{\text{m}}t}e^{-R_{2,\text{m}}^{*}t},\# \end{array}$$

and the signal parameters reported by Sati et al (2013), also listed in Table B1, unless stated otherwise. We then simulated two cases and varied: i. the myelin water signal fraction, $f_{\text{m}}$, from 0.1 to 0.2; or ii. the intra-axonal relaxation rate, $R_{2,\text{a}}^{*}$, from 15 to 35 s^−1^. Fig. 1(2)A and B show the frequency difference and the magnitude of the signal for each case, respectively.

We then fitted the 3-pool model assuming $R_{2,\text{a}}^{*}=0$, to test the influence of this assumption on the estimated myelin water signal fraction. Fig. 1C shows the estimated MW signal fraction plotted against the input value. We also tested the effect of the assumption $R_{2,\text{a}}^{*}=R_{2,\text{e}}^{*}$ in Fig. 1D. Finally, Fig. 2C shows MW signal fraction values estimated under the assumption of $R_{2,\text{a}}^{*}=0$ and plotted against the input $R_{2,\text{a}}^{*}$-value. Colormaps in Fig. B1, Fig. B2 reflect the variable input values for $f_{\text{m}}$ and $R_{2,\text{a}}^{*}$, respectively.

Overall, though a small bias can be observed in the estimated MW signal fractions for the given extra-axonal R2*, such bias is of the same magnitude for all input MW signal fractions and is smaller than the calculated CV values for this segment of the CC, suggesting that any bias introduced by possible differences in axonal R2* between groups is unlikely to have affected results in our study.

## References

[b0005] Aboitiz F., Scheibel A.B., Fisher R.S., Zaidel E. (1992). Fiber composition of the human corpus callosum. Brain Res..

[b0010] Alonso-Ortiz E., Levesque I.R., Pike G.B. (2018). Impact of magnetic susceptibility anisotropy at 3 T and 7 T on T2*-based myelin water fraction imaging. NeuroImage.

[b0015] Alonso-Ortiz E., Levesque I.R., Pike G.B. (2018). Multi-gradient-echo myelin water fraction imaging: Comparison to the multi-echo-spin-echo technique. Magn. Reson. Med..

[b0020] Andrews S.C., Domínguez J.F., Mercieca E.-C., Georgiou-Karistianis N., Stout J.C. (2015). Cognitive interventions to enhance neural compensation in Huntington’s disease. Neurodegenerative Disease Manage..

[b0025] Aylward E.H., Nopoulos P.C., Ross C.A., Langbehn D.R., Pierson R.K., Mills J.A., Johnson H.J., Magnotta V.A., Juhl A.R., Paulsen J.S. (2011). Longitudinal change in regional brain volumes in prodromal Huntington disease. J. Neurol. Neurosurg. Psychiatry.

[b0030] Ferrari Bardile C., Garcia-Miralles M., Caron N.S., Rayan N.A., Langley S.R., Harmston N., Rondelli A.M., Teo R.T.Y., Waltl S., Anderson L.M., Bae H.-G., Jung S., Williams A., Prabhakar S., Petretto E., Hayden M.R., Pouladi M.A. (2019). Intrinsic mutant HTT-mediated defects in oligodendroglia cause myelination deficits and behavioral abnormalities in Huntington disease. Proc. Natl. Acad. Sci..

[b0035] Baron‐Cohen S., Wheelwright S., Hill J., Raste Y., Plumb I. (2001). The “Reading the Mind in the Eyes” Test Revised Version: A study with normal adults, and adults with asperger syndrome or high-functioning autism. J. Child Psychol. Psychiatry.

[b0040] Bartzokis G., Cummings J., Perlman S., Hance D.B., Mintz J. (1999). Increased basal ganglia iron levels in Huntington disease. Arch. Neurol..

[b0045] Bartzokis G., Lu P.H., Tishler T.A., Fong S.M., Oluwadara B., Finn J.P., Huang D., Bordelon Y., Mintz J., Perlman S. (2007). Myelin breakdown and iron changes in Huntington’s disease: Pathogenesis and treatment implications. Neurochem. Res..

[b0050] Beaulieu C. (2002). The basis of anisotropic water diffusion in the nervous system—A technical review. NMR Biomed..

[b0055] Berthold C.-H., Nilsson I., Rydmark M. (1983). Axon diameter and myelin sheath thickness in nerve fibres of the ventral spinal root of the seventh lumbar nerve of the adult and developing cat. J. Anat..

[b0060] Birkl C., Birkl-Toeglhofer A.M., Endmayr V., Höftberger R., Kasprian G., Krebs C., Haybaeck J., Rauscher A. (2019). The influence of brain iron on myelin water imaging. NeuroImage.

[b0065] Bourbon-Teles J., Bells S., Jones D.K., Coulthard E., Rosser A., Metzler-Baddeley C. (2019). Myelin Breakdown in Human Huntington’s Disease: Multi-Modal Evidence from Diffusion MRI and Quantitative Magnetization Transfer. Non-Invasive MRI Windows Brain Inflam..

[b0070] Bydder M., Larkman D.J., Hajnal J.V. (2002). Combination of signals from array coils using image-based estimation of coil sensitivity profiles. Magn. Reson. Med..

[b0075] Casella C., Lipp I., Rosser A., Jones D.K., Metzler‐Baddeley C. (2020). A critical review of white matter changes in Huntington’s Disease. Mov. Disord..

[b0080] Cercignani M., Giulietti G., Dowell N.G., Gabel M., Broad R., Leigh P.N., Harrison N.A., Bozzali M. (2017). Characterizing axonal myelination within the healthy population: A tract-by-tract mapping of effects of age and gender on the fiber g-ratio. Neurobiol. Aging.

[b0085] Chan K.-S., Marques J.P. (2020). Multi-compartment relaxometry and diffusion informed myelin water imaging – Promises and challenges of new gradient echo myelin water imaging methods. NeuroImage.

[b0090] Ciarmiello A., Cannella M., Lastoria S., Simonelli M., Frati L., Rubinsztein D.C., Squitieri F. (2006). Brain white-matter volume loss and glucose hypometabolism precede the clinical symptoms of Huntington’s disease. J. Nucl. Med..

[b0095] Connor J.R., Menzies S.L., Martin S.M.S., Mufson E.J. (1990). Cellular distribution of transferrin, ferritin, and iron in normal and aged human brains. J. Neurosci. Res..

[b0100] Crawford, H. E., Hobbs, N. Z., Keogh, R., Langbehn, D. R., Frost, C., Johnson, H., Landwehrmeyer, B., Reilmann, R., Craufurd, D., Stout, J. C., Durr, A., Leavitt, B. R., Roos, R. A. C., Tabrizi, S. J., Scahill, R. I., & TRACK-HD Investigators. (2013). Corpus callosal atrophy in premanifest and early Huntington’s disease. Journal of Huntington’s Disease, 2(4), 517–526. https://doi.org/10.3233/JHD-130077. 10.3233/JHD-130077 25062736

[b0105] Cronin M.J., Wang N., Decker K.S., Wei H., Zhu W.-Z., Liu C. (2017). Exploring the origins of echo-time-dependent quantitative susceptibility mapping (QSM) measurements in healthy tissue and cerebral microbleeds. Neuroimage.

[b0110] Dayalu P., Albin R.L. (2015). Huntington disease: Pathogenesis and treatment. Neurol. Clin..

[b0115] De Paepe A.E., Sierpowska J., Garcia-Gorro C., Martinez-Horta S., Perez-Perez J., Kulisevsky J., Rodriguez-Dechicha N., Vaquer I., Subira S., Calopa M., Muñoz E., Santacruz P., Ruiz-Idiago J., Mareca C., de Diego-Balaguer R., Camara E. (2019). White matter cortico-striatal tracts predict apathy subtypes in Huntington’s disease. NeuroImage: Clin..

[b0120] De Santis S., Drakesmith M., Bells S., Assaf Y., Jones D.K. (2014). Why diffusion tensor MRI does well only some of the time: Variance and covariance of white matter tissue microstructure attributes in the living human brain. Neuroimage.

[b0125] Della Sala S., Gray C., Baddeley A., Wilson L. (1997). Visual Patterns Test: A test of short-term visual recall. Thames Valley Test Company.

[b0130] Dexter D.T., Carayon A., Javoy-Agid F., Agid Y., Wells F.R., Daniel S.E., Lees A.J., Jenner P., Marsden C.D. (1991). Alterations in the levels of iron, ferritin and other trace metals in Parkinson’s disease and other neurodegenerative diseases affecting the basal ganglia. *Brain: A*. J. Neurol..

[b0135] Di Paola, M., Luders, E., Cherubini, A., Sanchez-Castaneda, C., Thompson, P. M., Toga, A. W., Caltagirone, C., Orobello, S., Elifani, F., Squitieri, F., & Sabatini, U. (2012). Multimodal MRI analysis of the corpus callosum reveals white matter differences in presymptomatic and early Huntington’s disease. Cerebral Cortex (New York, N.Y.: 1991), 22(12), 2858–2866. https://doi.org/10.1093/cercor/bhr360. 10.1093/cercor/bhr360 22223853

[b0140] Di Paola M., Phillips O.R., Sanchez-Castaneda C., Di Pardo A., Maglione V., Caltagirone C., Sabatini U., Squitieri F. (2014). MRI measures of corpus callosum iron and myelin in early Huntington’s disease. Hum. Brain Mapp..

[b0145] Du Y.P., Chu R., Hwang D., Brown M.S., Kleinschmidt-DeMasters B.K., Singel D., Simon J.H. (2007). Fast multislice mapping of the myelin water fraction using multicompartment analysis of T decay at 3T: A preliminary postmortem study. Magn. Resonance Med..

[b0150] Dumas E.M., van den Bogaard S.J.A., Ruber M.E., Reilman R.R., Stout J.C., Craufurd D., Hicks S.L., Kennard C., Tabrizi S.J., van Buchem M.A., van der Grond J., Roos R.A.C. (2012). Early changes in white matter pathways of the sensorimotor cortex in premanifest Huntington’s disease. Hum. Brain Mapp..

[b0155] Ecker U.K.H., Oberauer K., Lewandowsky S. (2014). Working memory updating involves item-specific removal. J. Mem. Lang..

[b0160] Erickson K., Prakash R., Kim J., Sutton B., Colcombe S., Kramer A. (2009). Top-down attentional control in spatially coincident stimuli enhances activity in both task-relevant and task-irrelevant regions of cortex. Behav. Brain Res..

[b0165] Faria A.V., Ratnanather J.T., Tward D.J., Lee D.S., van den Noort F., Wu D., Brown T., Johnson H., Paulsen J.S., Ross C.A., Younes L., Miller M.I. (2016). Linking white matter and deep gray matter alterations in premanifest Huntington disease. NeuroImage: Clin..

[b0170] Fréchet M. (1957). Sur la distance de deux lois de probabilité. Comptes Rendus Hebdomadaires des seances de l academie des Sci..

[b0175] Freeman G.L. (1940). The relationship between performance level and bodily activity level. J. Exp. Psychol..

[b0180] Gareau P.J., Rutt B.K., Karlik S.J., Mitchell J.R. (2000). Magnetization transfer and multicomponent T2 relaxation measurements with histopathologic correlation in an experimental model of MS. J. Magn. Resonan. Imag..

[b0185] Goldstein A., Covington B.P., Mahabadi N., Mesfin F.B. (2020). Neuroanatomy.

[b0190] Gomez-Tortosa E., MacDonald M.E., Friend J.C., Taylor S.A.M., Weiler L.J., Cupples L.A., Srinidhi J., Gusella J.F., Bird E.D., Vonsattel J.-P., Myers R.H. (2001). Quantitative neuropathological changes in presymptomatic Huntington’s disease. Ann. Neurol..

[b0195] Gregory S., Crawford H., Seunarine K., Leavitt B., Durr A., Roos R.A.C., Scahill R.I., Tabrizi S.J., Rees G., Langbehn D., Orth M. (2018). Natural biological variation of white matter microstructure is accentuated in Huntington’s disease. Hum. Brain Mapp..

[b0200] Grieve S.M., Williams L.M., Paul R.H., Clark C.R., Gordon E. (2007). Cognitive Aging, Executive Function, and Fractional Anisotropy: A Diffusion Tensor MR Imaging Study. Am. J. Neuroradiol..

[b0205] Guttmann C.R.G., Jolesz F.A., Kikinis R., Killiany R.J., Moss M.B., Sandor T., Albert M.S. (1998). White matter changes with normal aging. Neurology.

[b0210] Harsan L.A., Poulet P., Guignard B., Steibel J., Parizel N., Loureiro de Sousa P., Boehm N., Grucker D., Ghandour M.S. (2006). Brain dysmyelination and recovery assessment by noninvasive in vivo diffusion tensor magnetic resonance imaging. J. Neurosci. Res..

[b0215] Henkelman R.M., Huang X., Xiang Q.-S., Stanisz G.J., Swanson S.D., Bronskill M.J. (1993). Quantitative interpretation of magnetization transfer. Magn. Reson. Med..

[b0220] Henkelman R.M., Stanisz G.J., Graham S.J. (2001). Magnetization transfer in MRI: A review. NMR Biomed..

[b0225] Hopfinger J.B., Buonocore M.H., Mangun G.R. (2000). The neural mechanisms of top-down attentional control. Nat. Neurosci..

[b0230] Huang B., Wei W., Wang G., Gaertig M.A., Feng Y., Wang W., Li X.-J., Li S. (2015). Mutant huntingtin downregulates myelin regulatory factor-mediated myelin gene expression and affects mature oligodendrocytes. Neuron.

[b0235] Jellinger K., Paulus W., Grundke-Iqbal I., Riederer P., Youdim M.B.H. (1990). Brain iron and ferritin in Parkinson’s and Alzheimer’s diseases. J. Neural Transmission-Parkinson’s Disease and Dementia Section.

[b0240] Jin J., Peng Q., Hou Z., Jiang M., Wang X., Langseth A.J., Tao M., Barker P.B., Mori S., Bergles D.E., Ross C.A., Detloff P.J., Zhang J., Duan W. (2015). Early white matter abnormalities, progressive brain pathology and motor deficits in a novel knock-in mouse model of Huntington’s disease. Hum. Mol. Genet..

[b0245] Jokinen H., Ryberg C., Kalska H., Ylikoski R., Rostrup E., Stegmann M.B., Waldemar G., Madureira S., Ferro J.M., van Straaten E.C.W., Scheltens P., Barkhof F., Fazekas F., Schmidt R., Carlucci G., Pantoni L., Inzitari D., Erkinjuntti T. (2007). Corpus callosum atrophy is associated with mental slowing and executive deficits in subjects with age-related white matter hyperintensities: The LADIS Study. J. Neurol. Neurosurg. Psychiatry.

[b0250] Kennedy K.M., Raz N. (2009). Aging white matter and cognition: Differential effects of regional variations in diffusion properties on memory, executive functions, and speed. Neuropsychologia.

[b0255] Kirchner W.K. (1958). Age differences in short-term retention of rapidly changing information. J. Exp. Psychol..

[b0260] Kleban E., Gowland P., Bowtell R. (2021). Probing the myelin water compartment with a saturation-recovery, multi-echo gradient-recalled echo sequence. Magn. Reson. Med..

[b0265] Kleban E., Tax C.M.W., Rudrapatna U.S., Jones D.K., Bowtell R. (2020). Strong diffusion gradients allow the separation of intra- and extra-axonal gradient-echo signals in the human brain. NeuroImage.

[b0270] Krishnamoorthy K., Lee M. (2014). Improved tests for the equality of normal coefficients of variation. Comput. Stat..

[b9005] Laule C., Vavasour I.M., Moore G.R.W. (2004). Water content and myelin water fraction in multiple sclerosis. J. Neurol..

[b9010] Laule C., Leung E., Li D.K. (2006). Myelin water imaging in multiple sclerosis: quantitative correlations with histopathology. Mult. Scler. J..

[b0275] Laule C., Kozlowski P., Leung E., Li D.K.B., Mackay A.L., Moore G.R.W. (2008). Myelin water imaging of multiple sclerosis at 7 T: Correlations with histopathology. NeuroImage.

[b0280] Lee H., Nam Y., Lee H.-J., Hsu J.-J., Henry R.G., Kim D.-H. (2018). Improved three-dimensional multi-echo gradient echo based myelin water fraction mapping with phase related artifact correction. NeuroImage.

[b0285] Lenzi D., Conte A., Mainero C., Frasca V., Fubelli F., Totaro P., Caramia F., Inghilleri M., Pozzilli C., Pantano P. (2007). Effect of corpus callosum damage on ipsilateral motor activation in patients with multiple sclerosis: A functional and anatomical study. Hum. Brain Mapp..

[b0290] Li X.u., Harrison D.M., Liu H., Jones C.K., Oh J., Calabresi P.A., van Zijl P.C.M. (2016). Magnetic susceptibility contrast variations in multiple sclerosis lesions. J. Magn. Reson. Imag..

[b0295] X. Li P. van Gelderen P. Sati J.A. de Zwart D.S. Reich J.H. Duyn Detection of demyelination in multiple sclerosis by analysis of [Formula: See text] relaxation at 7 T NeuroImage. Clinical 7 2015 709 714 PubMed. 10.1016/j.nicl.2015.02.021. 10.1016/j.nicl.2015.02.021 PMC4593862 26594617

[b0300] Lintl P., Braak H. (1983). Loss of intracortical myelinated fibers: A distinctive age-related alteration in the human striate area. Acta Neuropathol..

[b0305] MacKay A.L., Laule C. (2016). Magnetic resonance of myelin water: An in vivo marker for myelin. Brain Plast..

[b0310] Mackay A., Whittall K., Adler J., Li D., Paty D., Graeb D. (1994). In vivo visualization of myelin water in brain by magnetic resonance. Magn. Reson. Med..

[b0315] Martenson R.E. (1992). Myelin.

[b0320] Marwick B., Krishnamoorthy K. (2019). cvequality: Tests for the equality of coefficients of variation from multiple groups. R Package Version.

[b0325] McColgan P., Gregory S., Razi A., Seunarine K.K., Gargouri F., Durr A., Roos R.A.C., Leavitt B.R., Scahill R.I., Clark C.A., Tabrizi S.J., Rees G. (2017). White matter predicts functional connectivity in premanifest Huntington’s disease. Ann. Clin. Transl. Neurol..

[b0330] McDonald S., Rushby J.A., Dalton K.I., Allen S.K., Parks N. (2018). The role of abnormalities in the corpus callosum in social cognition deficits after Traumatic Brain Injury. Soc. Neurosci..

[b0335] Mueller S.T., Piper B.J. (2014). The psychology experiment building language (PEBL) and PEBL test battery. J. Neurosci. Methods.

[b0340] Myers R.H., Vonsattel J.P., Paskevich P.A., Kiely D.K., Stevens T.J., Cupples L.A., Richardson E.P., Bird E.D. (1991). Decreased neuronal and increased oligodendroglial densities in huntington’s Disease Caudate Nucleus. J. Neuropathol. Exp. Neurol..

[b0345] Nam Y., Kim D.-H., Lee J. (2015). Physiological noise compensation in gradient-echo myelin water imaging. NeuroImage.

[b0350] Nam Y., Lee J., Hwang D., Kim D.-H. (2015). Improved estimation of myelin water fraction using complex model fitting. NeuroImage.

[b0355] Nasreddine Z.S., Phillips N.A., Bédirian V., Charbonneau S., Whitehead V., Collin I., Cummings J.L., Chertkow H. (2005). The montreal cognitive assessment, MoCA: A brief screening tool for mild cognitive impairment. J. Am. Geriatr. Soc..

[b0360] Nopoulos P.C. (2016). Huntington disease: A single-gene degenerative disorder of the striatum. Dial. Clin. Neurosci..

[b0365] Nunes D., Cruz T.L., Jespersen S.N., Shemesh N. (2017). Mapping axonal density and average diameter using non-monotonic time-dependent gradient-echo MRI. J. Magn. Reson..

[b0370] Pakkenberg B., Pelvig D., Marner L., Bundgaard M.J., Gundersen H.J.G., Nyengaard J.R., Regeur L. (2003). Aging and the human neocortex. Exp. Gerontol..

[b0375] Paulsen J.S. (2011). Cognitive impairment in huntington disease: Diagnosis and treatment. Curr. Neurol. Neurosci. Reports.

[b0380] Paulsen J.S., Langbehn D.R., Stout J.C., Aylward E., Ross C.A., Nance M., Guttman M., Johnson S., MacDonald M., Beglinger L.J., Duff K., Kayson E., Biglan K., Shoulson I., Oakes D., Hayden M. (2008). Detection of Huntington’s disease decades before diagnosis: The Predict-HD study. J. Neurol. Neurosurg. Psychiatry.

[b0385] Peters A. (2009). The effects of normal aging on myelinated nerve fibers in monkey central nervous system. Front. Neuroanat..

[b0390] Phillips O., Sanchez-Castaneda C., Elifani F., Maglione V., Di Pardo A., Caltagirone C., Squitieri F., Sabatini U., Di Paola M., Tang Y.-P. (2013). Tractography of the corpus callosum in Huntington’s Disease. PLoS One.

[b0395] Pierpaoli C., Basser P.J. (1996). Toward a quantitative assessment of diffusion anisotropy. Magn. Reson. Med..

[b0400] Radulescu C.I., Garcia-Miralles M., Sidik H., Ferrari Bardile C., Yusof N.A.B.M., Lee H.U., Ho E.X.P., Chu C.W., Layton E., Low D., De Sessions P.F., Pettersson S., Ginhoux F., Pouladi M.A. (2018). Manipulation of microbiota reveals altered myelination and white matter plasticity in a model of Huntington disease. BioRxiv.

[b0405] Rey-Mermet A., Gade M., Oberauer K. (2018). Should we stop thinking about inhibition? Searching for individual and age differences in inhibition ability. J. Exp. Psychol. Learn. Mem. Cogn..

[b0410] Roemer P.B., Edelstein W.A., Hayes C.E., Souza S.P., Mueller O.M. (1990). The NMR phased array. Magn. Reson. Med..

[b0415] Rubinsztein D.C., Leggo J., Coles R., Almqvist E., Biancalana V., Cassiman J.-J., Chotai K., Connarty M., Craufurd D., Curtis A. (1996). Phenotypic characterization of individuals with 30–40 CAG repeats in the Huntington disease (HD) gene reveals HD cases with 36 repeats and apparently normal elderly individuals with 36–39 repeats. Am. J. Hum. Genet..

[b0420] Ruocco H.H., Bonilha L., Li L.M., Lopes-Cendes I., Cendes F. (2008). Longitudinal analysis of regional grey matter loss in Huntington disease: Effects of the length of the expanded CAG repeat. J. Neurol. Neurosurg. Psychiatry.

[b0425] Sati P., van Gelderen P., Silva A.C., Reich D.S., Merkle H., de Zwart J.A., Duyn J.H. (2013). Micro-compartment specific T2* relaxation in the brain. NeuroImage.

[b0430] Schweser F., Deistung A., Lehr B.W., Reichenbach J.R. (2011). Quantitative imaging of intrinsic magnetic tissue properties using MRI signal phase: An approach to in vivo brain iron metabolism?. Neuroimage.

[b0435] Shaffer J.J., Ghayoor A., Long J.D., Kim R.-E.-Y., Lourens S., O’Donnell L.J., Westin C.-F., Rathi Y., Magnotta V., Paulsen J.S., Johnson H.J. (2017). Longitudinal diffusion changes in prodromal and early HD: Evidence of white-matter tract deterioration. Hum. Brain Mapp..

[b0440] Shmueli K., Dodd S.J., Li T.-Q., Duyn J.H. (2011). The contribution of chemical exchange to MRI frequency shifts in brain tissue. Magn. Reson. Med..

[b0445] S.J. Tabrizi D.R. Langbehn B.R. Leavitt R.A. Roos A. Durr D. Craufurd C. Kennard S.L. Hicks N.C. Fox R.I. Scahill B. Borowsky A.J. Tobin H.D. Rosas H. Johnson R. Reilmann B. Landwehrmeyer J.C. Stout TRACK-HD investigators. Biological and clinical manifestations of Huntington’s disease in the longitudinal TRACK-HD study: Cross-sectional analysis of baseline data The Lancet. Neurology 8 9 2009 791 801 10.1016/S1474-4422(09)70170-X. 10.1016/S1474-4422(09)70170-X PMC3725974 19646924

[b0450] S.J. Tabrizi R. Reilmann R.A.C. Roos A. Durr B. Leavitt G. Owen R. Jones H. Johnson D. Craufurd S.L. Hicks C. Kennard B. Landwehrmeyer J.C. Stout B. Borowsky R.I. Scahill C. Frost D.R. Langbehn TRACK-HD investigators. Potential endpoints for clinical trials in premanifest and early Huntington’s disease in the TRACK-HD study: Analysis of 24 month observational data The Lancet. Neurology 11 1 2012 42 53 10.1016/S1474-4422(11)70263-0. 10.1016/S1474-4422(11)70263-0 22137354

[b0455] Tabrizi S.J., Scahill R.I., Durr A., Roos R.A., Leavitt B.R., Jones R., Landwehrmeyer G.B., Fox N.C., Johnson H., Hicks S.L., Kennard C., Craufurd D., Frost C., Langbehn D.R., Reilmann R., Stout J.C., TRACK-HD Investigators (2011). Biological and clinical changes in premanifest and early stage Huntington’s disease in the TRACK-HD study: The 12-month longitudinal analysis. Lancet. Neurol..

[b0460] Tendler B.C., Bowtell R. (2019). Frequency difference mapping applied to the corpus callosum at 7T. Magn. Reson. Med..

[b0465] Teo R.T.Y., Hong X., Yu-Taeger L., Huang Y., Tan L.J., Xie Y., To X.V., Guo L., Rajendran R., Novati A., Calaminus C., Riess O., Hayden M.R., Nguyen H.P., Chuang K.-H., Pouladi M.A. (2016). Structural and molecular myelination deficits occur prior to neuronal loss in the YAC128 and BACHD models of Huntington disease. Hum. Mol. Genet..

[b0470] Thapaliya K., Vegh V., Bollmann S., Barth M. (2018). Assessment of microstructural signal compartments across the corpus callosum using multi-echo gradient recalled echo at 7 T. NeuroImage.

[b0475] van Gelderen P., de Zwart J.A., Lee J., Sati P., Reich D.S., Duyn J.H. (2012). Non-exponential T2* decay in White Matter. Magn. Reson. Med..

[b0480] van Gelderen P., Duyn J.H. (2019). White matter intercompartmental water exchange rates determined from detailed modeling of the myelin sheath. Magn. Reson. Med..

[b0485] Veraart J., Nunes D., Rudrapatna U., Fieremans E., Jones D.K., Novikov D.S., Shemesh N. (2020). Noninvasive quantification of axon radii using diffusion MRI. ELife.

[b0490] Veraart J., Raven E.P., Edwards L., Weiskopf N., Jones D.K. (2021). The variability of MR axon radii estimates in the human white matter. Hum. Brain Mapp..

[b0495] Wang, J., Zamar, R., Marazzi, A., Yohai, V., Salibian-Barrera, M., Maronna, R., Zivot, E., Rocke, D., Doug, M., Maechler, M., & Konis, K. (2009). Package ‘robust’. CRAN.

[b9000] Webb S., Munro C.A., Midha R., Stanisz G.J. (2003). Is multicomponent *T*_2_ a good measure of myelin content in peripheral nerve?. Magn. Reson. Med..

[b0500] Wechsler D. (1997). Wechsler Adult Intelligence Scale—Revised UK.

[b0505] Wharton, S., & Bowtell, R. (2012). Fiber orientation-dependent white matter contrast in gradient echo MRI. Proceedings of the National Academy of Sciences of the United States of America, 109(45), 18559–18564. https://doi.org/10.1073/pnas.1211075109. 10.1073/pnas.1211075109 PMC3494918 23091011

[b0510] Wharton S., Bowtell R. (2013). Gradient echo based fiber orientation mapping using R2* and frequency difference measurements. NeuroImage.

[b0515] Wheeler-Kingshott C.A.M., Cercignani M. (2009). About “axial” and “radial” diffusivities. Magn. Reson. Med..

[b0520] Wilkinson L. (1999). Statistical methods in psychology journals: Guidelines and explanations. Am. Psychol..

[b0525] Wisnieff C., Ramanan S., Olesik J., Gauthier S., Wang Y., Pitt D. (2015). Quantitative susceptibility mapping (QSM) of white matter multiple sclerosis lesions: Interpreting positive susceptibility and the presence of iron. Magn. Reson. Med..

[b0530] Wu D., Faria A.V., Younes L., Mori S., Brown T., Johnson H., Paulsen J.S., Ross C.A., Miller M.I. (2017). Mapping the order and pattern of brain structural MRI changes using change-point analysis in premanifest Huntington’s disease. Hum. Brain Mapp..

[b0535] Xu T., Foxley S., Kleinnijenhuis M., Chen W.C., Miller K.L. (2018). The effect of realistic geometries on the susceptibility-weighted MR signal in white matter. Magn. Reson. Med..

[b0540] Xu, T., Foxley, S., & Miller, K. (2015). Oligodendrocytes and the role of iron in magnetic susceptibility driven frequency shifts in white matter. Proceedings of the 23rd Annual Meeting of ISMRM, Toronto, Canada.

[b0545] Yablonskiy D.A., He X., Luo J., Sukstanskii A.L. (2014). Lorentz sphere versus generalized Lorentzian approach: What would lorentz say about it?. Magn. Reson. Med..

[b0550] Yin P., Liu Q., Pan Y., Yang W., Yang S., Wei W., Chen X., Hong Y., Bai D., Li X.-J., Li S. (2020). Phosphorylation of myelin regulatory factor by PRKG2 mediates demyelination in Huntington’s disease. EMBO Rep..

[b0555] Zecca L., Youdim M.B.H., Riederer P., Connor J.R., Crichton R.R. (2004). Iron, brain ageing and neurodegenerative disorders. Nat. Rev. Neurosci..

[b0560] Zhang J., Gregory S., Scahill R.I., Durr A., Thomas D.L., Lehericy S., Rees G., Tabrizi S.J., Zhang H. (2018). In vivo characterization of white matter pathology in premanifest huntington’s disease. Ann. Neurol..

